# Enamel pits and enamel developmental defects in tuberous sclerosis complex: A descriptive synthesis and pooled prevalence analysis

**DOI:** 10.4317/medoral.28247

**Published:** 2026-06-28

**Authors:** Hiroshi Nakagawa

**Affiliations:** 1 Tokushima University Hospital, Pediatric Dentistry, Japan

**Keywords:** Tuberous sclerosis complex, enamel pits, enamel developmental defects, systematic review, meta‑analysis.

## Abstract

**Background:**

Enamel pits and enamel developmental defects have been reported in patients with tuberous sclerosis complex (TSC), but prevalence estimates and phenotypic descriptions vary widely. A standardized synthesis has been lacking. Recent updates in TSC clinical reviews and dermatologic-dental guidelines highlight the diagnostic relevance of subtle oral findings, underscoring the need for an updated synthesis.

**Material and Methods:**

A systematic review with quantitative integration was conducted in accordance with PRISMA 2020 guidelines. Electronic databases were searched from inception to March 2026. Eligible studies reporting enamel pits or enamel developmental defects in TSC were reviewed, and pooled prevalence was calculated using a random‑effects model. Meta‑analytic procedures, including Forest and funnel plot generation, were performed using EZR (version 1.7). Heterogeneity was assessed using the I^2^ statistic, and subgroup analyses explored sources of variability. A phenotype‑oriented classification system was developed based on recurring enamel features. Representative clinical cases were included only as qualitative supplementary material. Recent literature on TSC diagnostic criteria and enamel defects in systemic or dermatologic conditions was additionally incorporated to contextualize methodological variability.

**Results:**

Seven studies involving 217 patients were included. Enamel pits and related defects were frequently reported. The pooled prevalence of enamel pits was 66%, with substantial heterogeneity (I^2^=77%). Individual estimates ranged from 0% in deciduous dentition to 100% in studies using staining or SEM. Funnel plot asymmetry suggested potential publication bias and small‑study effects. Variability was partly associated with dentition type, age, diagnostic criteria, and assessment methods. These findings align with contemporary evidence emphasizing that enamel abnormalities may be under‑recognized depending on examination methods and clinical context.

**Conclusions:**

This review synthesizes current evidence on enamel abnormalities in TSC and highlights considerable variability in reported prevalence. The proposed phenotype‑oriented classification provides a pragmatic descriptive framework, but findings should be interpreted cautiously due to methodological heterogeneity. Contemporary literature supports the diagnostic relevance of enamel pits as a recognized minor feature of TSC, reinforcing the need for standardized assessment.

## Introduction

Tuberous sclerosis complex (TSC) is a multisystem genetic disorder characterized by hamartomas affecting multiple organs, including the brain, skin, kidneys, and heart. Oral manifestations -particularly enamel developmental defects- have been recognized for decades; however, the literature remains inconsistent with respect to their reported prevalence, diagnostic criteria, and clinical interpretation.

Enamel pits are frequently described as characteristic findings in TSC, yet reported detection rates vary widely. This variability appears to be influenced by dentition type, patient age, and diagnostic methods used, complicating comparison across studies. Moreover, although numerous reports describe enamel defects in TSC, no unified framework exists to integrate historical and contemporary evidence or to guide standardized clinical assessment. Despite numerous reports, no prior review has quantitatively synthesized prevalence estimates using formal metaanalytic methods. In particular, no study has applied modern statistical tools such as EZR (a graphical interface for R) to evaluate pooled prevalence and publication bias. Addressing this gap is essential for clarifying the true frequency of enamel defects in TSC.

Recent updates in TSC diagnostic frameworks and dermatologic-dental guidelines emphasize the importance of subtle oral findings, including enamel pits, which are recognized as a minor diagnostic feature of TSC [1-11].  Additionally, recent clinical studies on developmental enamel defects in systemic and dermatologic conditions highlight the broader relevance of enamel abnormalities as indicators of altered ectodermal development [12].These developments underscore the need to reevaluate enamel findings in TSC using updated clinical perspectives.

A clearer understanding of enamel defect patterns may support earlier recognition of TSC in dental settings. Therefore, the aims of this study were to: Systematically synthesize available evidence on enamel developmental defects in TSC; examine sources of variability in reported detection rates across dentition types and diagnostic methods; and propose a clinically applicable, phenotypeoriented classification system grounded in systematic evidence synthesis.

## Materials and Methods

*Study design*

This study was designed as a systematic review with quantitative integration where feasible, informed by the PRISMA 2020 statement to enhance methodological transparency and reproducibility.

*Search strategy*

A comprehensive literature search was conducted using PubMed and Google Scholar from database inception to March 2026. The search strategy combined terms related to tuberous sclerosis complex and enamel abnormalities, including “tuberous sclerosis complex,” “enamel pits,” “enamel hypoplasia,” “dental anomalies,” and “developmental defects.”  

In addition, reference lists of relevant articles were manually screened to identify further eligible studies.

*Study selection*

The database search identified 47 records, and 3 additional records were identified through manual reference screening. After removal of 14 duplicate records, 36 records remained and were screened based on titles and abstracts.  

Of these, 22 records were excluded because they were not related to enamel defects in TSC, were nonclinical studies, or did not contain original data.  

Fourteen fulltext articles were assessed for eligibility. Following fulltext review, 7 articles were excluded due to lack of extractable enamelspecific data, inability to isolate data specific to TSC, or insufficient clinical description of enamel findings.  

Ultimately, 7 studies met the inclusion criteria and were included in the final synthesis.  

The study selection process is summarized in the PRISMA flow diagram (Figure 1).

*Eligibility criteria*

Studies were included if they met all of the following criteria: Human clinical studies; inclusion of patients diagnosed with tuberous sclerosis complex; reporting of enamel pits or enamel developmental defects with extractable prevalence or descriptive data.  

Reviews, editorials, conference abstracts, animal studies, and reports without enamelspecific clinical information were excluded.

*Quantitative synthesis*

Metaanalysis was performed using EZR (version 1.7; Saitama Medical Center, Jichi Medical University, Japan), which provides a userfriendly interface for R and is widely used for medical statistics.  

Event counts and total sample sizes were extracted from each study. Pooled prevalence was calculated using a randomeffects model (DerSimonian-Laird method). Ninetyfive percent confidence intervals were computed using the Wilson method.

Betweenstudy heterogeneity was quantified using the I² statistic. A Forest plot was generated to visualize individual and pooled prevalence estimates. Potential publication bias and smallstudy effects were assessed using a funnel plot.

Recent literature on TSC diagnostic criteria and enamel defects in systemic or dermatologic conditions was additionally reviewed to contextualize methodological variability and strengthen interpretation [9-12].

*Clinical case observations*

Representative clinical cases from Tokushima University Hospital were reviewed solely as qualitative supplementary material to illustrate enamel phenotypes described in the literature. These cases were not included in the systematic review dataset and had no influence on prevalence estimates or quantitative comparisons.

## Results

*Study selection*

The literature search identified 47 records through database searching and 3 additional records through manual screening of reference lists. After removal of 14 duplicate records, 36 records were screened by title and abstract. Of these, 22 records were excluded for not meeting the inclusion criteria.  

Fourteen fulltext articles were assessed for eligibility, and 7 articles were excluded due to lack of extractable enamelspecific data, inability to isolate data specific to patients with tuberous sclerosis complex, or insufficient clinical description of enamel findings.  

Ultimately, 7 studies were included in the final synthesis.  

The study selection process is illustrated in the PRISMA flow diagram (Figure 1).

*Prevalence of enamel pits*

Across included studies, enamel pits were consistently reported as the most frequent enamel abnormality in TSC (Figure 2, Table 1) [1-5].  

In permanent dentition, reported prevalence ranged from 48% to 100%, depending on diagnostic methods employed [1,3-5,7].  

In contrast, detection rates in deciduous teeth were markedly lower, often reported as 0%, suggesting differences related to developmental timing (Table 2) [2,7].

*Influence of diagnostic method*

Detection rates varied substantially by assessment method.  

Scanning electron microscopy revealed micropits not visible during routine clinical examination [1], while staining techniques increased detection compared with visual inspection alone [3,4].  

Studies relying solely on visual examination tended to report lower prevalence [5,7].

*Other enamel developmental defects*

Incisal edge defects were frequently obscured by tooth wear, particularly in older patients [7].  

Tooth wear due to attrition or trauma further masked developmental defects.  

Generalized enamel hypoplasia was less common but was reported more frequently in patients with TSC than in control populations [5,6].

*Proposed phenotypeoriented classification*

Based on recurring enamel phenotypes identified across studies [1-7], a structured classification system was developed, comprising: Enamel pits, incisal edge defects, tooth wear, and generalized enamel hypoplasia.  

*Metaanalysis of enamel pit prevalence*

A randomeffects metaanalysis was performed using EZR to quantitatively synthesize enamel pit prevalence across the seven included studies.  

The pooled prevalence of enamel pits was 66%, with substantial betweenstudy heterogeneity (I²=77%).  

Individual study estimates ranged from 0% in deciduous dentition to 100% in studies using staining or scanning electron microscopy.  

The Forest plot (Figure 3) illustrates the variability in prevalence across studies.

Funnel plot inspection (Figure 4) demonstrated visible asymmetry, suggesting potential publication bias and smallstudy effects in addition to methodological heterogeneity. These findings are consistent with contemporary reports indicating that enamel abnormalities may be underdetected depending on examination methods and clinical context [11,12].

## Discussion

This systematic review integrates historical and contemporary evidence to clarify the spectrum of enamel developmental defects associated with TSC. As highlighted in the Introduction, enamel pits have long been described as characteristic findings in TSC [1,3-5], yet reported prevalence has varied widely across studies. The present synthesis confirms this inconsistency and demonstrates that much of the variability arises from methodological and biological factors identified in the results.

Across the included studies, enamel pits were consistently the most frequently reported abnormality, particularly in permanent dentition [1-5,7]. The markedly lower detection rates in deciduous teeth align with developmental timing differences and support the notion that enamel pit formation is more strongly associated with the maturation period of permanent teeth [2,7].

The metaanalysis conducted using EZR confirmed substantial heterogeneity among studies, underscoring the need for standardized diagnostic criteria and calibrated assessment methods. Funnel plot asymmetry suggests that publication bias or smallstudy effects may influence the current evidence base, although methodological variability remains a major contributing factor.

Methodological heterogeneity emerged as a major determinant of reported prevalence. Studies employing scanning electron microscopy or staining techniques detected substantially more pits than those relying solely on visual inspection [1,3,4]. Agerelated factors, including tooth wear and restorative history, further complicated the identification of incisal edge defects and generalized enamel hypoplasia [5-7].

Based on recurring enamel phenotypes synthesized from the included studies, we developed a phenotypeoriented classification system comprising enamel pits, incisal edge defects, tooth wear, and generalized enamel hypoplasia [1-7]. This framework is intended to harmonize descriptive terminology rather than serve as a diagnostic tool. Its clinical utility remains to be validated, and future studies should assess its reproducibility and predictive value using standardized criteria and calibrated examiners.

Recent updates in TSC diagnostic frameworks emphasize that enamel pits constitute one of the recognized minor diagnostic features of TSC, reinforcing the importance of systematic oral examination in affected individuals [8-11].Furthermore, recent clinical studies on developmental enamel defects in systemic and dermatologic conditions highlight that enamel abnormalities may reflect broader ectodermal involvement, supporting their relevance in syndromic evaluation [12]. Incorporating these contemporary perspectives strengthens the clinical context of our findings and underscores the need for standardized dental assessment in TSC.

These findings also underscore the important role of dentists in the early recognition of TSC. Because enamel pits are readily observable during routine dental examinations, they may serve as accessible clinical markers prompting timely referral for systemic evaluation [3-5].

*Several limitations warrant consideration*

First, the search strategy was limited to PubMed and Google Scholar, which may have excluded relevant studies indexed in other databases.

Second, few included studies incorporated control groups, limiting the ability to determine the specificity of enamel defects for TSC.

Third, contemporary guidelines highlight the need for standardized dental examination protocols in TSC, including the use of adjunctive methods such as disclosing agents to enhance pit detection [11].

In summary, this review consolidates available evidence and proposes a structured framework for describing enamel phenotypes in TSC. While enamel pits remain a prominent and potentially informative clinical feature, methodological differences across studies limit the precision of prevalence estimates. Continued refinement of diagnostic approaches and external validation of phenotypebased classifications will be essential to improving clinical recognition and research comparability.

## Conclusions

Enamel developmental defects, particularly enamel pits, are commonly reported in patients with TSC, although reported prevalence varies widely across studies. Differences related to dentition type and diagnostic methodology highlight the need for standardized assessment approaches. Recent clinical guidance underscores the diagnostic relevance of enamel pits as a recognized minor feature of TSC, reinforcing the importance of systematic dental evaluation in affected individuals [8-11]. The proposed phenotypeoriented classification system provides a pragmatic framework for consistent description, but further welldesigned studies are required to refine prevalence estimates and validate its clinical utility. 

## Figures and Tables

**Table 1:** Table 1: Summary of major studies on enamel pits and enamel defects in tuberous sclerosis complex. Characteristics of seven key studies, including sample size, diagnostic methods, prevalence findings, and notable quotations. Detection rates varied widely depending on dentition type, assessment technique (visual inspection, staining, or scanning electron microscopy), and study design.

**Author (Year)**	**Sample size / population**	**Diagnostic method**	**Prevalence / key findings**	**Notable quotations**
Hoff *et al*., 1975	6 TSC patients	Clinical exam + SEM	100% enamel pits	“The pitshaped enamel defects seem to be pathognomonic of the disease.”
Lygidakis & Lindenbaum, 1987	49 TSC, 68 relatives, 50 controls	Visual inspection	71% in typical TSC	“One parent and one halfsib… were found to have multiple enamel pits.”
Mlynarczyk, 1991	50 TSC, 250 controls	Staining + visual exam	100% in TSC; 7% in controls	“The incidence of enamel pitting… was 100%.”
Sampson *et al*., 1992	23 permanenttooth patients, 6 deciduousonly	Visual inspection	48% (permanent), 0% (deciduous)	“None were seen in six patients with deciduous teeth.”
Flanagan *et al*., 1997	10 TSC, 20 relatives, 25 controls	Staining + probing	100% in TSC; 70% had ≥15 pits	“70% of TS cases had more than 14 pits per person.”
Araújo *et al*., 2011	13 TSC (family study)	Clinical exam	53.8% enamel pits	“Dental enamel pits were the most common features…”
Korporowicz *et al*., 2020	60 TSC, 60 controls	Clinical exam	41.7% enamel hypoplasia; 6.2× more frequent than controls	“Enamel hypoplasia was observed 6.2 times more often…”

**Table 2:** Table 2: Comparison of enamel pit detection between deciduous and permanent dentition in tuberous sclerosis complex. Detection rates of enamel pits across dentition types, highlighting consistently lower detection in deciduous teeth and higher prevalence in permanent dentition, particularly when staining or scanning electron microscopy methods were used. Notes summarize methodological factors influencing detection.

**Study**	**Dentition type**	**Sample size**	**Detection rate of enamel pits**	**Notes**
Sampson *et al.,* 1992	Permanent teeth	23 patients	48%	Visual inspection only
Sampson *et al.,* 1992	Deciduous teeth	6 patients	0%	“None were seen in six patients with deciduous teeth.”
Araújo *et al*., 2011	Mixed dentition	13 patients	53.8%	Age variation affected detection
Korporowicz *et al*., 2020	Mixed dentition	60 patients	41.7% (hypoplasia)	Wear often masked incisal defects
Hoff *et al.,* 1975	Permanent teeth	6 patients	100%	SEM revealed micropits not visible clinically
Mlynarczyk, 1991	Adult permanent teeth	50 patients	100%	Staining method increased detection

**Figure 1:**
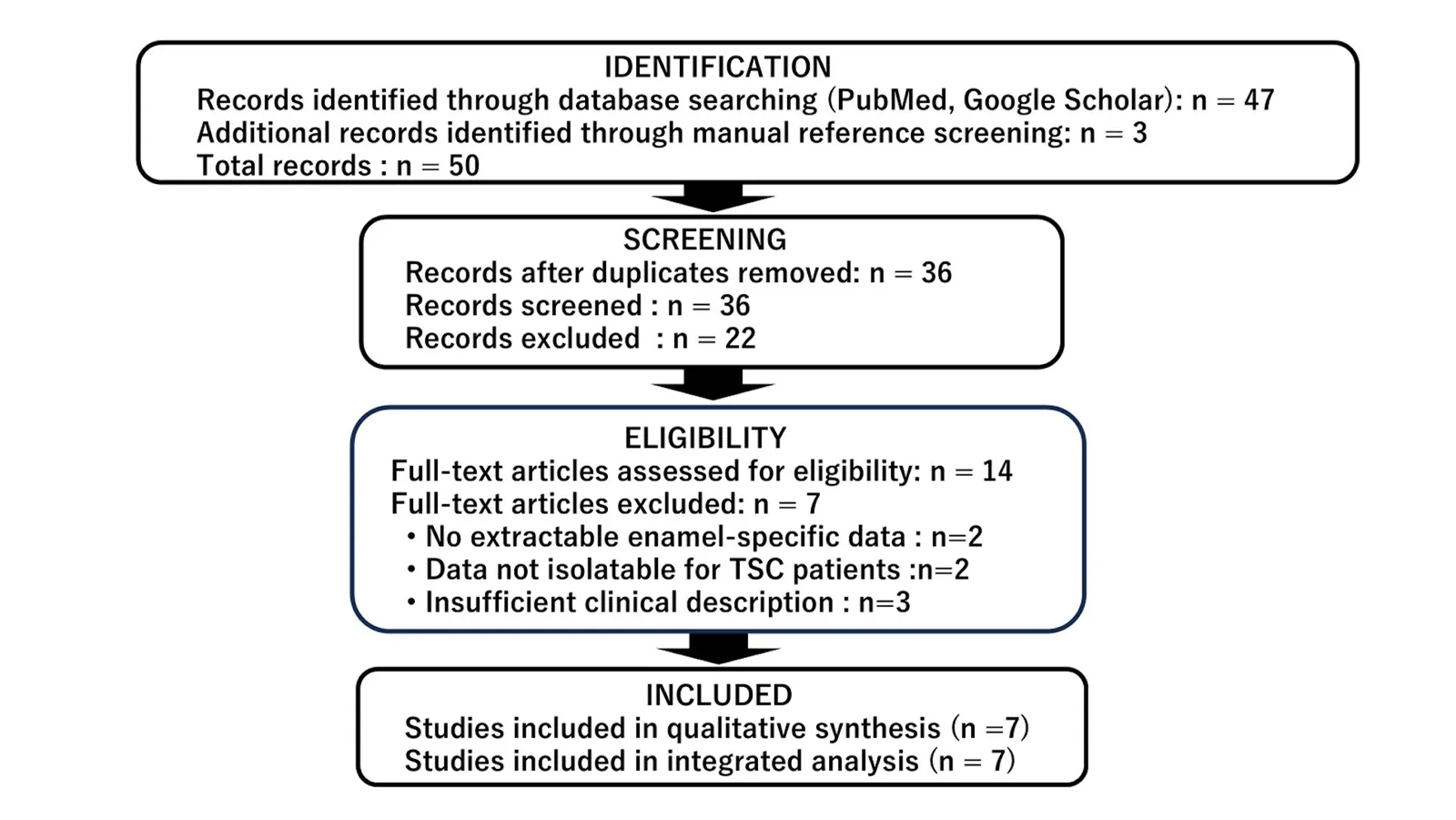
Fig. 1: PRISMA 2020 flow diagram summarizing the study selection process. The diagram illustrates the identification, screening, eligibility assessment, and final inclusion of studies in accordance with PRISMA 2020 guidelines. A total of 50 records were identified (47 through database searching and 3 through manual reference screening). After removal of 14 duplicates, 36 records were screened, of which 22 were excluded based on titles and abstracts. Fourteen full‑text articles were assessed for eligibility, and 7 were excluded due to lack of extractable enamel‑specific data (n=2), inability to isolate data specific to patients with tuberous sclerosis complex (n=2), or insufficient clinical description (n=3). Ultimately, 7 studies were included in both the qualitative synthesis and integrated analysis.

**Figure 2:**
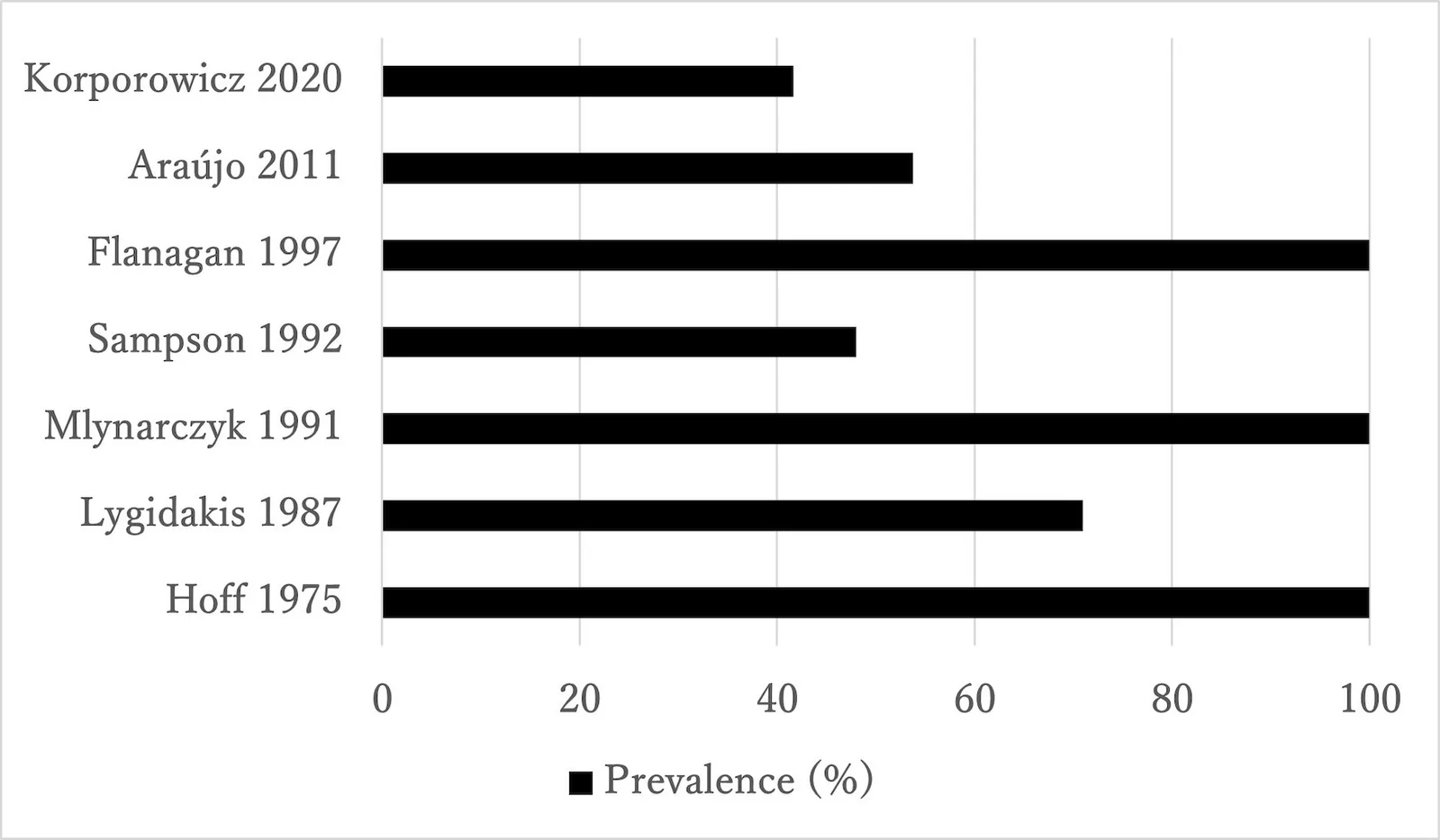
Fig. 2: Reported prevalence of enamel pits across studies included in the systematic review. This figure summarizes the prevalence of enamel pits reported in seven studies of patients with tuberous sclerosis complex. Detection rates varied widely, ranging from 0% in deciduous dentition to 100% in studies using staining techniques or scanning electron microscopy. Variability reflects differences in dentition type, diagnostic methodology, and study design.

**Figure 3:**
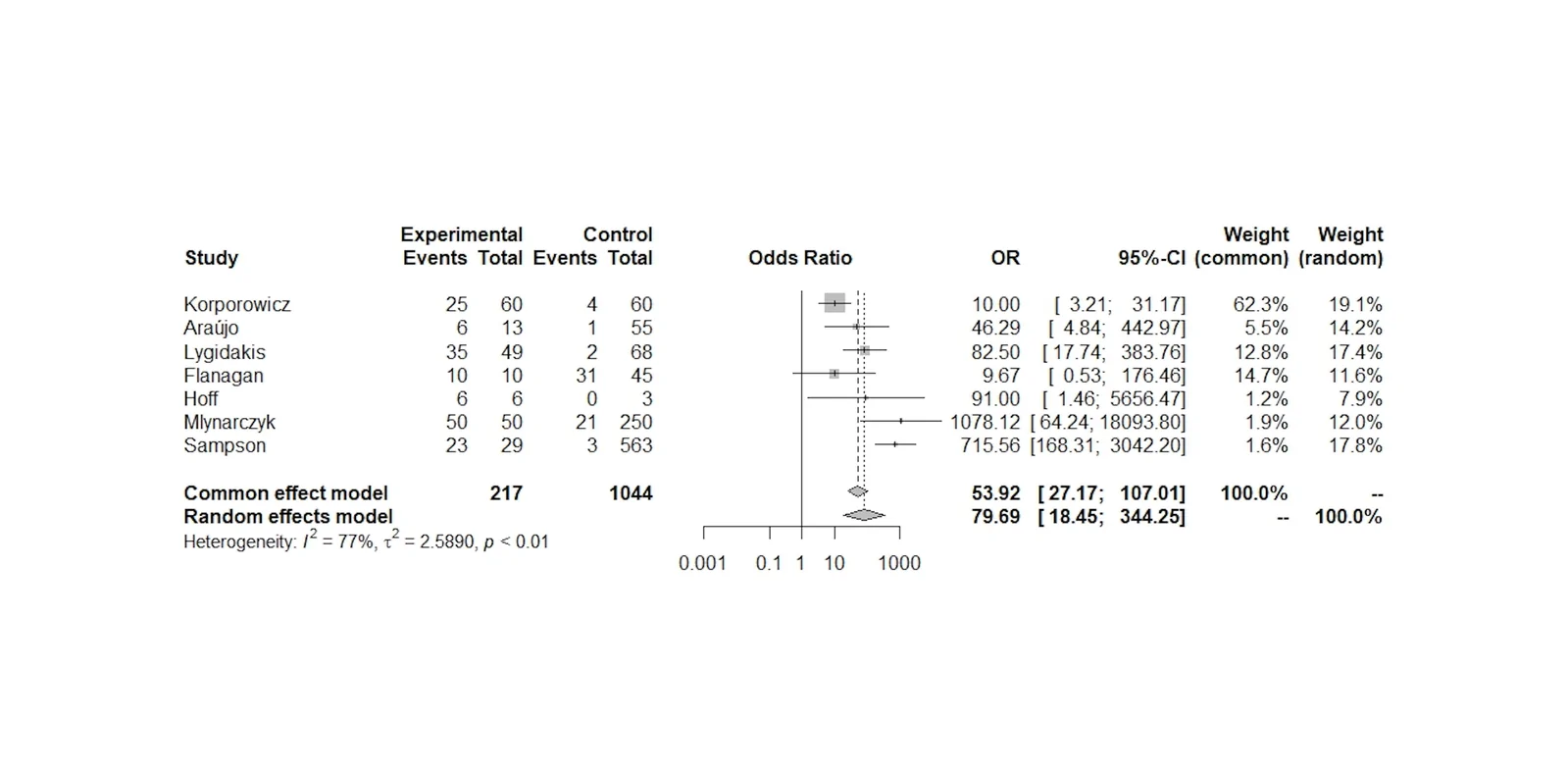
Fig. 3: Forest plot of enamel pit prevalence in patients with tuberous sclerosis complex. Pooled prevalence estimates were calculated using a random‑effects model based on data extracted from seven eligible studies. Individual study proportions are shown with 95% confidence intervals. The size of each square reflects the study weight. The diamond represents the pooled prevalence estimate. Meta‑analysis was performed using EZR (version 1.7), a graphical user interface for R.

**Figure 4:**
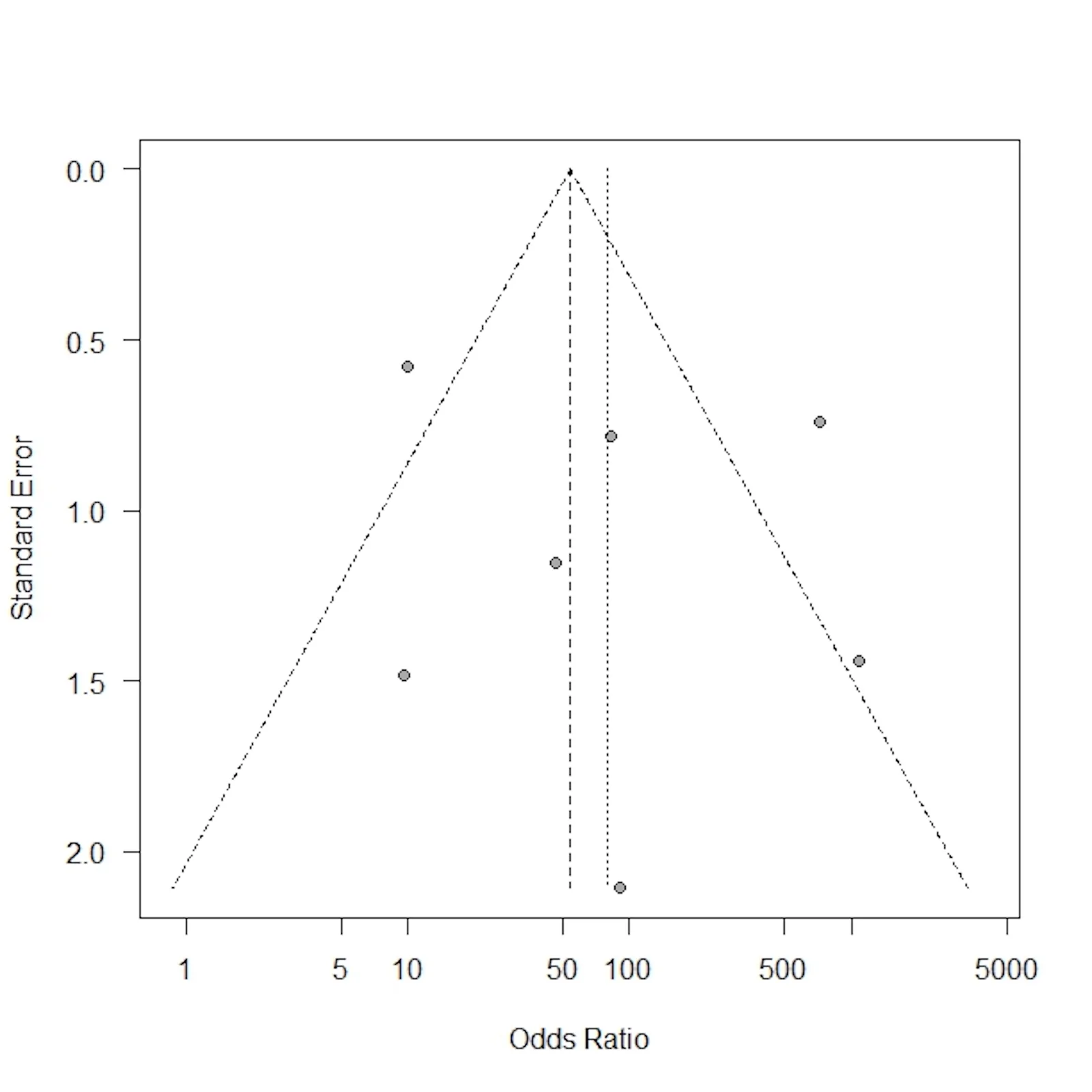
Fig. 4: Funnel plot assessing publication bias and small‑study effects in studies reporting enamel pit prevalence in tuberous sclerosis complex. The vertical line indicates the pooled effect estimate from the random‑effects model, and the diagonal lines represent pseudo 95% confidence limits. Visual inspection demonstrates asymmetry, suggesting potential publication bias and methodological heterogeneity. Funnel plot generation and statistical calculations were performed using EZR (version 1.7).

## Data Availability

None.
